# Atomic fluorescence determination of mercury in fresh water ecosystems

**DOI:** 10.1155/S1463924695000113

**Published:** 1995

**Authors:** Randy Knox, William R. Kammin, David Thomson

**Affiliations:** Washington State Department of Ecology 7411 Beach Drive Port Orchard Washington 98366-8204 USA

## Abstract

This paper reports on an investigation into determining nanogram/l quantities of mercury in marine and fresh water matrices using a cold vapour generation of mercury, followed by fluorescence detection. Samples were prepared for analysis using a free bromine oxidation technique. A high efficiency gas-liquid separator was used to enhance the detection of mercury. For fresh water, typical method detection limits (MDL) were determined at less than 1 nanogram/l (ng/l). For near shore seawater, the MDL was 1.2 ng/l. Method spikes, which were performed at 20 ng/l, showed mean recoveries within US EPA Contract Laboratory Protocol (CLP) acceptance criteria. System blanks averaged 0.12 ng/l, and recoveries of NIST 1641c diluted to 29.4 ng/l averaged 93.4%. A number of local rivers and streams were sampled, and mercury was determined. All results to date indicate mercury levels below the US EPA chronic water quality criteria for mercury.


Journal of Automatic Chemistry, Vol. 17, No. 2 (March–April 1995), pp. 65–71

### Atomic fluorescence determination of mercury in fresh water ecosystems

**Randy Knox, William R. Kammin and David Thomson**

*Washington State Department of Ecology, 7411 Beach Drive, Port Orchard, Washington 98366-8204, USA*

*This paper reports on an investigation into determining nanogram/l quantities of mercury in marine and fresh water matrices using a cold vapour generation of mercury, followed by fluorescence detection. Samples were prepared for analysis using a free bromine oxidation technique. A high efficiency gas-liquid separator was used to enhance the detection of mercury. For fresh water, typical method detection limits (MDL) were determined at less than 1 nanogram/l (ng/l). For near shore seawater, the MDL was 1.2 ng/l. Method spikes, which were performed at 20 ng/l, showed mean recoveries within US EPA Contract Laboratory Protocol (CLP) acceptance criteria. System blanks averaged 0.12 ng/l, and recoveries of NIST 1641c diluted to 29·4 ng/l averaged 93·4%. A number of local rivers and streams were sampled, and mercury was determined. All results to date indicate mercury levels below the US EPA chronic water quality criteria for mercury.*

### Introduction

Significant concerns remain regarding mercury contamination in aqueous ecosystems. The United States Environmental Protection Agency (US EPA) has advanced water quality criteria [1] for the protection of organisms native to water environments. The chronic water quality criterion for mercury in fresh water ecosystems is 12 nanograms/litre (ng/l), and the mercury chronic criterion for salt water is 25 ng/l. These extremely low criteria present significant problems for the currently approved US EPA regulatory methods (245.1–5 and SW-846 7470-7471) in that these methods, which are based on atomic absorption techniques, generally produce instrument detection limits between 30–200 ng/l. This level of detection is insufficient to quantify mercury levels in many borderline impacted waters.

The use of atomic fluorescence for the determination of mercury was first reported by Thomson and Reynolds in 1971 [2]. Since then, several authors [3–5] have described enhancements to the technique that have reduced formal instrument detection limits (IDL) for the fluorescence technique to the 1–10 ng/l range. The authors of this paper report on the use of atomic fluorescence detection coupled with a high efficiency gas-liquid separator, the combination of which has reduced the instrument detection limit for mercury to less than 1 ng/l. A bromine-based sample digestion technique, which results in 'cleaner' sample preparation, is also described. The potassium bromide and potassium bromate digestion reagents used in this method to produce bromine are purified by heating at 300 °C to remove mercury. The acids used in the procedure are purified by sub-boiling distillation in a Teflon still. Using these purified reagents results in undetectable levels of mercury (<0·5 ng/l) in the procedural blanks. In contrast, the currently approved US EPA Methods 245·1 and SW-846 7470-7471 use relatively large amounts of reagents that are difficult to purify completely of mercury contamination.

Another innovation in this method is the use of disposable polyethylene terephthalate (PETG) bottles for mercury sample preparation. The use of disposable sample preparation bottles reduces the problems of mercury contamination when preparation bottles are reused (reuse is common in environmental laboratories that use the currently approved US EPA regulatory methods).

### Method summary

The method used in this work for the determination of mercury is based on detection of mercury by atomic fluorescence at 253·7 nanometers (nm) excitation and emission wavelength. Samples are digested before analysis with a mixture of potassium bromate and bromide in hydrochloric acid [6]. This mixture, at ambient temperatures, forms free bromine, which oxidizes both organic and inorganic forms of mercury to $Hg^{++}$ [6 and 7].

A fluorescence mercury analysis system (Merlin, supplied by P. S. Analytical Ltd, Sevenoaks, Kent, UK) was used for mercury determinations. Oxidized sample, or blank solution and $SnCl_2$ reagent, are combined in the mixing valve of the instrument reduction apparatus. $Hg^{++}$ is reduced to elemental mercury, which is carried in the sample stream. The carrier mixture containing elemental mercury is then directed into a high efficiency gas-liquid separator. A stream of fine argon bubbles sweeps elemental mercury out of solution into the gaseous phase; the gas stream containing mercury vapour is then passed through a drying tube to remove water vapour; then dry vapour is swept into the instrument's fluorescence detector. A mercury vapour lamp with a characteristic wavelength of 253·7 nm is used to excite mercury atoms in the vapour. The intensity of emission at the same wavelength is measured and used to quantitate mercury.

### Sampling considerations

### Sample collection and handling

Water samples are collected in precleaned Teflon bottles; they are then preserved by acidifying to pH < 2 with

0142-0453/95 $10.00 © 1995 Taylor & Francis Ltd.

about 3 ml mercury free nitric acid per litre. Samples are refrigerated at 4 ± 2 °C upon receipt at the laboratory. Preparation for analysis is carried out within seven days of sample receipt and final analysis completed within an additional week.

Polyethylene terepthalate G copolymer (PETG) 125 ml and 250 ml bottles (Nalgene brand No. 2019 or equivalent) are used for digestion of samples and standards. All new or washed bottles are stored filled with blank solution (non-decolorized) in order to oxidize any mercury-containing compounds. Blank solution is formed in the bottles by adding 25 ml 1 + 1 HCl to about 200 ml of American Society for Testing and Materials (ASTM) type II water in the bottles, followed by 5 ml of 0.1N potassium bromate/bromide solution. Bottles are filled to about 1 cm from the top and cap. Yellow colour (free bromine) was to be stable until use. For 125 ml bottles, the above amounts need to be halved. The solution is decolorized with 0·15 ml of 12% hydroxylamine hydrochloride (0·075 ml for 125 ml bottles). It is then stirred and discarded; the bottles are rinsed three times with a small amount ASTM type II water. The recapped bottles are then ready for use. Cleaning should be carried out either in a cleanroom or in an area with an extremely low mercury background.

### Reagent preparation

Reagent preparation is crucial to the success of these low-level determinations to ensure that all plasticware is properly precleaned, and that all reagents are prepared in a consistent manner in a mercury-free area.

All reagents must be suitable for mercury or trace metal analysis. Trace metals grade hydrochloric and nitric acids should be purified by sub-boiling distillation in a Teflon still. ASTM type II, or better, DI water should be used for this.

*2·0 μg/ml stock mercury standard*

A 1000 ml volumetric flask is rinsed, and then 200 ml $H_2O$ and 20 ml $HNO_3$ added; 2·0 ml of a suitable 1000 ppm mercury standard (SPEX Industries, Inc., Edison, NJ, or equivalent) is measured into the volumetric flask and diluted to volume with Type II water, and mixed.

*Intermediate standard preparation*

25 ml of 2·0 μg/ml stock is diluted to 500 ml in 2% $HNO_3$: this gives a 100 μg/l mercury solution.

Working standards are made from a further intermediate dilution. A 1 ml to 100 ml dilution in 2% $HNO_3$ of 100 μg/l stock gives a 1·0 μg/l standard. This working standard is then further diluted to give Hg standards of 0·002 to 0·05 μg/l. The 1·0 μg/l standard should be prepared daily as used.

*Spiking solution 4 μg/l*

4 ml of 100 μg/l intermediate standard is added to a 100 ml volumetric flask; then 2·0 ml $HNO_3$ is added and diluted to volume with Type II water.

*National Institute of Standards and Technology (NIST) 1641C stock solution*

10 ml of NIST 1641C standard is pipetted into a rinsed 100 ml volumetric which contains about 50 ml $H_2O$ and 2 ml of $HNO_3$; it is then diluted to volume. This diluted solution is 147 μg/ml in mercury; it is further diluted by pipetting 4 ml of diluted solution into a 100 ml flask and diluted to volume with 2% nitric acid to give a 5·88 μg/l standard.

*0·2N potassium bromate*

Dissolve 1·39 g potassium bromate (ACS reagent grade) in 250 ml ASTM Type II water. Potassium bromate is purified by heating overnight at 300 °C. Stored in a sealed bottle.

*0·2N potassium bromide*

5·95 g potassium bromide (ACS reagent grade) is dissolved in a 250 ml volumetric flask in ASTM Type II water and diluted to volume. Potassium bromide is purified by heating overnight at 300 °C. This should be stored in a sealed bottle.

*0·1N potassium bromate/bromide reagent*

Equal volumes of 0·2N potassium bromate and 0·2N potassium bromide are mixed. This reagent should be prepared fresh daily.

*12% hydroxylamine HCl solution*

12 g of hydroxylamine hydrochloride, $NH_2OH \cdot HCl$ is dissolved in 100 ml Type II water.

*Stannous chloride ($SnCl_2 \cdot 2H_2O$) 2% in 4% HCl*

20 ± 0·5 g of $SnCl_2 \cdot 2H_2O$ is weighed and added to a 1000 ml flask containing 40 ± 1 ml concentrated hydrochloric acid. This is heated gently, and stirred until dissolved; it is then diluted to mark with $H_2O$. About 1 l/min of argon is bubbled through reagent for 30 minutes prior to use in order to remove any traces of mercury.

*Blank solution*

32 ml $HNO_3$ + 100 ml HCl were added to a 2 l volumetric; 1500 ml DI water was added. 40 ml 0·1N bromate/bromide reagent is added; then the solution is diluted to the mark and mixed. It is decolourized with 1·2 ml 12% Hydroxylamine HCl. *Note*: Use subboiled purified $HNO_3$ and HCl should be used in blank solution.

### Experimental

Analyses were performed on a Merlin mercury system (P. S. Analytical, Sevenoaks, Kent, UK; see table 1) consisting of an automated mercury vapour generator with a high efficiency gas-liquid separator, a magnesium perchlorate drying tube, a Merlin fluorescence detector, and a controlling computer equipped with Touchstone software.

Blank solution, sample solution and $SnCl_2$ reductant solution are pumped in the three channels of the peristaltic pump (figure 1). A mixing valve combines $SnCl_2$ solution with blank or sample solution. Flows are determined by the pump tubing sizes, red (Questron Corp., Princeton, NJ, No. Z008T101) for nominal 8 ml/min flow and blue (Questron Corp., No. Z005T101) for about 4 ml/min flow. Argon gas at about 24 PSI is supplied as a carrier gas to the gas-liquid separator and as a sheath gas to the fluorescence detector to improve detector performance.

The drying tube (Leeman Instruments, Lowell, MA, No. 120-00067) used was approximately 15 cm by 1·5 cm. The ends of the tube were loosely packed with quartz wool. The tube was packed before each use with granular magnesium perchlorate (Leeman Instruments No. 020-1119). The drying tube worked well and greatly reduced carry-over of water into the fluorescence detector which had previously been a problem.

The high efficiency gas-liquid separator (Questron Instruments, Princeton, NJ, No. 10.101C) is illustrated in figure 2. The high efficiency performance results from the fine bubbles formed by the sintered plate; these bubbles are efficient in sweeping mercury from solution and result in a factor of two enhancement in sensitivity compared with the standard gas-liquid separator used with this instrument. Lower gas flow rates are used with this

*Table 1. Instrument parameters for PSA Merlin Mercury analyser.*

| *Liquid flows* | *ml/min* | *Gas flows* | *ml/min* |
|---|---|---|---|
| Sample | 8 | Sheath | 130 |
| Blank | 8 | Carrier | 90 |
| $SnCl_2$ | 4 | | |
| *Time* | *Seconds* | *Merlin Detector* | |
| Delay | 10 | Range | 1,000 |
| Rise | 99 | Fine | 4* |
| Measure | 48 | Integration | 1/4 s |
| Memory | 99 | | |
| Total | 256 (4·3 min) | | |

*Varied as needed for 50 ng/l standard at 0·75 full scale.

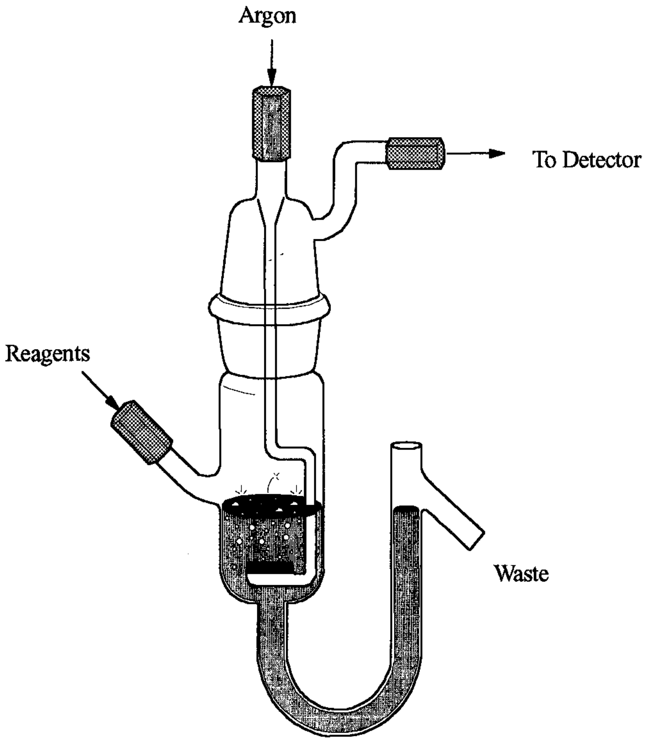

*Figure 2. An improved gas/liquid separator for mercury determinations.*

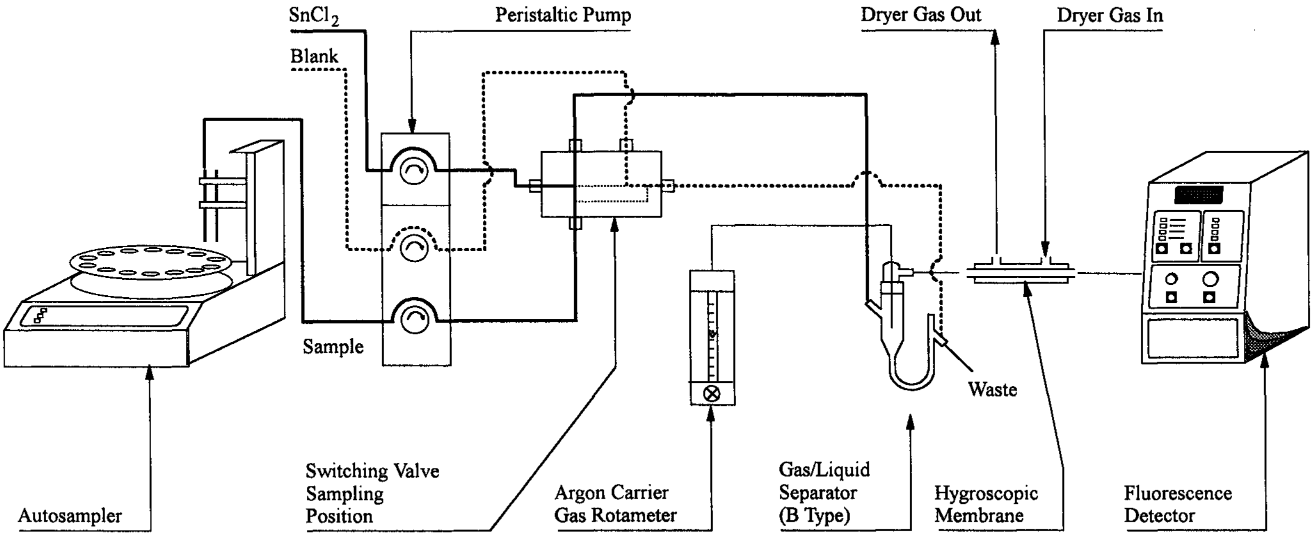

*Figure 1.*

separator as opposed to the standard separator, resulting in less dilution. These reduced flows also reduce moisture carry-over into the detector. The larger volume of the improved separator is a compromise which increases the time needed to rinse out the system, system memory time, and the time required after sample introduction for the signal to approach its maximum value (rise time). Greater efficiency results from finer argon bubbles being formed by sintered plate than are formed by tip used in standard separator. This results in more contact of gas and solution and increases likelihood of mercury transfer to gaseous phase producing greater efficiency of mercury removal per volume of argon. Greater solution-volume of cell also promotes transfer. However, this increase in volume also increases time to flush the system and thus the time of analysis.

*Sample and standard solution preparation*

Procedures for standard and sample preparation should be performed in an area determined to be free from mercury contamination. Standards for low level fluorescence work are prepaired by taking 10·0, 5·0, 2·0, 1·0, 0·4, and 0 ml aliquots of the 1·0 μg/l stock with a rinsed pipette; and then placed in 250 ml precleaned and rinsed PETG bottles. Each standard is diluted to 200 g to give 0·050, 0·020, 0·010, 0·005, and 0·002 μg/l standards; 25 ml 1 + 1 HCl, followed by 5 ml of 0·1 N potassium bromate/bromide solution, is added to each bottle. Then 20 ml of type II water is added to make a total 250 ml of prepared standard. Standards bottles should be kept sealed at all times when standard or reagents are not being added, to prevent absorption of atmospheric mercury.

To prepare samples, 100 ml of sample is added to tared precleaned 125 ml PETG media bottle. Any spike needed (0·50 ml of spiking solution for a 20 ng/l spike) is then added. Similarly, the laboratory control sample (LCS) is prepared by pipetting 1·0 ml of 5·88 μg/l 1641C solution into a 250 ml precleaned bottle and diluting to 200 g (29·4 ng/l); a procedure blank is similarly prepared with 200 ml of water. To each 100 ml of solution, 12·5 ml of 1 + 1 hydrochloric acid is added; this is followed with 2·5 ml 0·1N bromate/bromide solution. If the solution maintains a yellow colour, 10 ml water (20 ml for 200 ml original volumes) is added. However, if the solution decolourizes, an additional 2·5 ml of 0·1N bromate/bromide solution should be added and the added water volume reduced to 7·5 ml. If differing levels of digestion solution are added, a blank with the same reagent levels should be prepared.

Digested samples must stand for at least 1 h prior to analysis. After digestion, samples can be held up to seven days before analysis (seven days is recommended as the holding time in EPA Method 245·7).

*Analytical methodology*

The instrument was set up with the flows and conditions described in table 1 and allowed to stabilize for about 1 h. Sample and standard bottles were uncapped and an aliquot of 0·075 ml of 12% M/V hydroxylamine hydrochloride per 100 ml original solution was added to decolourize solution in each bottle. Bottles were recapped, swirled, and stored closed until analysis. Instrument response was checked by analysing the 50 ng/l standard and adjusting the fine response to give a peak of about 0·75 full scale.

*Calibration*

A calibration curve was established by running a single determination of each standard starting with the zero standard. Each standard was uncapped just before analysis. The instrument software then performs linear regression on the mercury response/concentration data. A correlation coefficient greater than or equal to 0·9985 was required to validate instrument calibration.

Calibration was verified with the NIST 1641C reference material. Acceptable response was ±15% of theoretical value; blank response was also verified and found acceptable if the response was below 1 ng/l. Calibration and blank response were re-verified every 10 samples and at the close of the run as per US EPA Contract Lab Protocol (CLP) requirements [8].

Typical detector response is shown in figure 3 for a 50 ng/l standard, a 2 ng/l standard, and a blank. Each standard was processed through the entire digestion procedure. Sample baseline response is measured in the earlier part of the curve. The curve approaches a maximum around 100 s. Peak height response is measured during a 48 s period when the response is close to maximum. The curve then returns to baseline before another determination is initiated. The total analysis time is approximately 4·3 min. Slightly more time is required to return to baseline after higher level samples. The response of a 2 ng/l standard is clearly distinguishable from the blank response.

A calibration curve from 0–50 ng/l is shown in figure 4. Regression linearity has generally been excellent, with all determined regressions having linear correlation coefficients at 0·9985 or greater.

Results on digested verification standard are listed in table 2. Recovery, as a percentage of theoretical, ranged from 85 to 104% for a 29·4 ng/l standard over the study period. Results for digestion blanks range from −0·37 to 0·47 ng/l for all runs over the study period (see table 3). The average determined blank value ± blank standard deviation is 0·12 ± 0·25 ng/l.

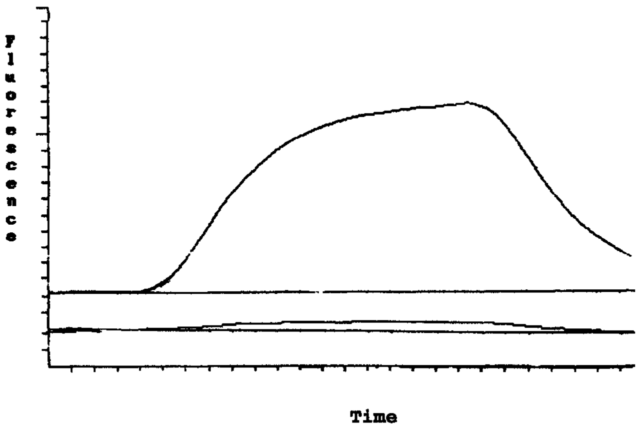

*Figure 3. From bottom, response of blank, 2 ng/l standard and 50 ng/l standard.*

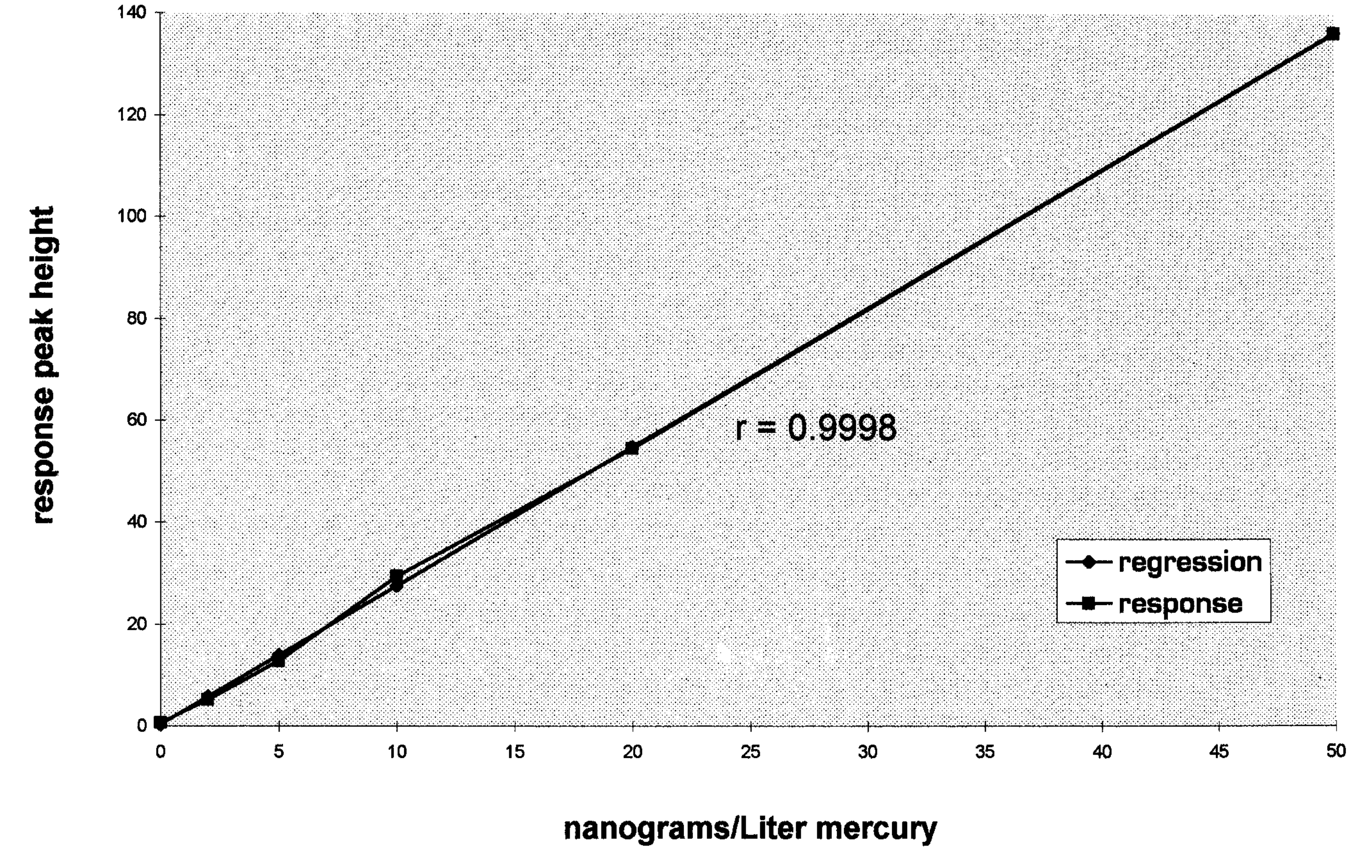

*Figure 4. Mercury fluorescence concentration response—typical calibration curve.*

*Table 2. Calibration verification standard results—CVAF/bromine oxidation method—standard is NIST 1641c diluted to 29·4 ng/l.*

| Run No. | Date | Results (ng/l) | | | |
|---|---|---|---|---|---|
| 1 | 5/20/94 | 28·71 | 25·66 | 28·71 | 26·44 |
| 2 | 5/24/94 | 27·84 | 26·65 | 26·25 | 26·02 |
| 3 | 5/27/94 | 28·6 | 27·77 | 27·08 | 26·56 |
| 4 | 6/2/94 | 27·2 | 27·42 | 28·34 | |
| 5 | 6/16/94 | 27·95 | 27·91 | 28·12 | |
| 6 | 6/29/94 | 26·23 | 25·04 | | |
| 7 | 7/19/94 | 28·58 | 30·66 | | |
| 8 | 7/28/94 | 28·68 | 29·19 | | |
| | True value | 29·4 | | | |
| | Mean | 27·57 | | | |
| | SD | 1·3 | | | |
| | % recovery | 93·8 | | | |

*Table 3. Procedural blank determinations—CVAF/bromine oxidation method.*

| Run No. | Date | Results (ng/l) | | | |
|---|---|---|---|---|---|
| 1 | 5/9/94 | −0·01 | 0·48 | −0·32 | 0·19 |
| 2 | 5/20/94 | 0·32 | 0·39 | 0·25 | 0·25 |
| 3 | 5/24/94 | 0·09 | 0·20 | 0·09 | 0·42 |
| 4 | 5/27/94 | −0·15 | −0·23 | −0·23 | −0·06 |
| 5 | 6/2/94 | 0·30 | 0·26 | 0·22 | |
| 6 | 6/16/94 | −0·20 | −0·37 | −0·03 | |
| 7 | 6/29/94 | 0·40 | 0·44 | | |
| 8 | 7/19/94 | 0·47 | 0·16 | | |
| 9 | 7/28/94 | 0·03 | 0·00 | | |
| | Mean | 0·12 | | | |
| | SD | 0·25 | | | |

*Method detection limit (MDL)*

MDLs have been determined in two sample matrices, laboratory tapwater and unfiltered, nearshore Manchester, Washington seawater. The published US EPA MDL procedure was used as guidance for MDL determination [9].

Data for an MDL determinations for tapwater are shown in table 4. The mean concentration determined was 0·56 ± 0·11 ng/l with the MDL determined to be 0·34 ng/l. The MDL was also determined on two other non-consecutive days to be 0·43 ng/l and 0·60 ng/l. For fresh water, Manchester Environmental Laboratory has established a reporting limit of 1 ng/l for this method.

Data are shown in table 5 for a determination of an MDL for Manchester seawater. The sample was not filtered, and particulates may contribute to sampling problems and raise the MDL. Filtering of seawater was not tested. If particulates which were somewhat difficult to suspend contained mercury and were not uniformly suspended, an increase in variability could result. The mean concentration was 3·17 ± 0·40 ng/l for a determined MDL of 1·2 ng/l.

*Table 4. MDL data—fresh water matrix—CVAF/bromine oxidation method (ng/l).*

| *Determination No.* | *Result* |
|---|---|
| Labwater 1 | 0·56 |
| Labwater 2 | 0·43 |
| Labwater 3 | 0·43 |
| Labwater 4 | 0·56 |
| Labwater 5 | 0·56 |
| Labwater 6 | 0·56 |
| Labwater 7 | 0·60 |
| Mean | 0·56 |
| SD | 0·11 |
| MDL | 0·34 |

*Table 5. MDL data—seawater matrix—CVAF/bromine oxidation method (ng/l).*

| *Determination No.* | *Result* |
|---|---|
| Seawater 1 | 3·38 |
| Seawater 2 | 3·21 |
| Seawater 3 | 3·84 |
| Seawater 4 | 3·34 |
| Seawater 5 | 2·87 |
| Seawater 6 | 2·66 |
| Seawater 7 | 2·87 |
| Mean | 3·17 |
| SD | 0·40 |
| MDL | 1·2 |

The MDLs presented here are at least one order of magnitude lower than the water quality criteria for fresh water and seawater. These levels were determined without the additional expense and difficulty of doing gold amalgamation concentration of mercury prior to analysis.

### Results—real world samples

Analytical results from a number of water samples are listed in table 6. Results for spike recoveries on some of these samples are listed in table 7. Spike levels used were generally 20 ng/l. All results are within ±25% except for the 2 ng/l duplicate spike on Manchester seawater. In this case the spike level of 2 ng/l was very close to the instrument detection limit where more noise in the spike recovery data will normally be observed. The reliability of the entire analytical and sampling protocol was further demonstrated when selected samples were re-run after several weeks' storage. There was no significant change in results for these samples (table 6).

The MDL, recovery, and blank data presented in this paper demonstrate that it is possible to obtain reliable ng/l level fresh water mercury data at levels less than a tenth of the EPA chronic water quality criteria. These data also indicate that levels of mercury in these selected Washington waters are generally well below the chronic water quality criteria for mercury.

Data from earlier papers [6 and 7] have demonstrated the effectiveness of bromine oxidation for releasing mercury from a number of matrices. It is significant that this new method is proving to be robust and is performed at room temperature. Also, it is possible to obtain the required MDLs needed without the additional step and expense of amalgamation concentration.

*Table 6. Mercury in Washington State rivers—bromine oxidation CVAF determination.*

| *Site* | *Result* | *Rerun** | *% of original* |
|---|---|---|---|
| Columbia River—Northport | 0·36 | | |
| Columbia River—Vernita | 0·69 | | |
| Columbia River—Umatilla | 1·46 | | |
| Columbia River—The Dalles | 1·20 | 0·97 | 81 |
| Spokane River—Stateline Br. | 0·81 | | |
| Sanpoil River | 9·77 | 10·8 | 111 |
| Palouse River—Hopper | 1·68 | 1·72 | 102 |
| Nisqually River | 1·09 | | |
| Deschutes River | 1·07 | | |
| Fife Ditch | 6·38 | 6·82 | 107 |
| Green River—Kanaskat | 0·46 | | |
| Cedar River—Maplewood | 0·42 | | |
| Issaquah Creek | 0·50 | | |
| Units—ng/l | | | |

*Samples rerun 26–29 days after original determination, storage in Teflon, 4°C.

*Table 7. Spike recovery data for bromine oxidation/CVAF method.*

| *Spike #* | *Date* | *Spiking level (ng/l)* | *Sample number* | *% Recovery spike* | *% Recovery spike dup.* |
|---|---|---|---|---|---|
| 1 | 5/9/94 | 5 | Labwater | 108 | |
| 2 | 5/20/94 | 20 | 198415 | 95 | 87 |
| 3 | 5/24/94 | 20 | 208437 | 89 | 80 |
| 4 | 5/27/94 | 20 | 218118 | 107 | 95 |
| 5 | 6/2/94 | 20 | 218122 | 92 | 90 |
| 6 | 6/2/94 | 2 | Seawater | 114 | 126 |
| 7 | 6/16/94 | 20 | 238010 | 97 | 102 |
| 8 | 7/19/94 | 20 | 276165 | 113 | 106 |
| 9 | 7/28/94 | 20 | 296064 | 102 | 100 |
| 10 | 7/28/94 | 20 | 296120 | 100 | 98 |

Future work will include the continued use of this method to characterize low level waters and work on exploring the use of bromine oxidation as a method of sample preparation for mercury in fresh water and marine sediments.

### References

1. EPA, *Quality Criteria for Water*, U.S. Environmental Protection Agency. Office of Water Regulations and Standards. EPA 440/5-86-001 (1986).
2. Thompson, K. C. and Reynolds, G. C., *Analyst* **96** (1971), 771.
3. Stockwell, P. and Godden, R. *Journal of Analytical Atomic Spectroscopy*, **4** (1989), 301.
4. Kammin, W. and Knox, R., *Environmental Laboratory* (August/September 1992).
5. Potter, B. *et al.*, US EPA Method 245·7, Revision 1·1 (1994) Environmental Monitoring and Systems Laboratory, Cincinnati, Ohio.
6. *Yorkshire Water Methods of Analysis*, 5th edn (1988).
7. Farey, B., Nelson, L. and Rolph, M., *Analyst*, **103** (1978), 656.
8. US EPA Contract Laboratory Program Statement of Work, ILM03.0.
9. Federal Register (26 October 1984), 198.

